# Editorial: Changing trends of precision medicine in diabetes

**DOI:** 10.3389/fmed.2025.1499934

**Published:** 2025-01-21

**Authors:** Hafiz Muhammad Asif, Allah Nawaz, Muhammad Daniyal

**Affiliations:** ^1^Faculty of Medicine and Allied Health Sciences, University College of Conventional Medicine, The Islamia University of Bahawalpur, Bahawalpur, Pakistan; ^2^Section on Integrated Physiology and Metabolism, Joslin Diabetes Center, Harvard Medical School, Boston, MA, United States; ^3^Department of Pediatrics, Division of Hematology and Oncology, Cancer Institute, Penn State College of Medicine, Hershey, PA, United States

**Keywords:** diabetes, precision medicine, type 1 and type 2 diabetes, DKD, Mendelian randomization analysis

## Introduction

Diabetes research has entered a transformative era with the advent of precision medicine, a paradigm that tailors treatment and prevention strategies to individual patient characteristics. This special Research Topic showcases a collection of six manuscripts that illuminate the evolving landscape of precision medicine in diabetes, highlighting innovative approaches that promise to enhance patient outcomes. As the global burden of diabetes continues to rise, it becomes imperative to move beyond the traditional “one-size-fits-all” treatment model. Precision medicine in diabetes focuses on understanding the genetic, environmental, and lifestyle factors that contribute to disease onset and progression. By leveraging advances in genomics, metabolomics, and big data analytics, researchers can identify biomarkers that predict treatment responses, thereby facilitating personalized interventions. As we embark on this journey through the evolving landscape of precision medicine in diabetes, our goal is to explore recent developments and uncover the therapeutic potential they hold for the future.

The manuscripts featured in this Research Topic explore various aspects of precision medicine in diabetes, including type 1 and type 2 diabetes (T1D and T2D, respectively). One of the key manuscripts in this collection presents a candidate composite biomarker that can guide treatment decisions for patients with diabetic kidney disease (DKD). Notably, Jones et al. have identified a composite biomarker that can help determine the most effective treatment options for patients with DKD, specifically differentiating the benefits of renin-angiotensin system inhibitors (RASi) alone and in combination with other treatment. Utilizing data from the PROVALID study, the researchers used machine learning techniques to identify parameters that predict the clinical benefits of adding a second treatment to RASi. Patients receiving the combination therapy had a significant improvement in estimated glomerular filtration rate (ΔeGFR), implying that personalized treatment approaches could significantly enhance patient outcomes. Thus, insights from this and other similar studies leveraging large-data are pivotal in refining treatment protocols, ensuring that patients receive the most effective therapies while minimizing adverse effects.

Neves et al. identified the different insulin infusion systems (IIS)-associated factors that significantly influence the diabetes management. In recent years, insulin infusion sets have become an essential tool for managing diabetes, particularly for patients using insulin pumps. However, despite their widespread use, these devices are not without complications. Research shows that infusion site issues, including inflammation, occlusions, and infections, occur in a significant percentage of users, sometimes leading to severe outcomes such as hyperglycemia or diabetic ketoacidosis if not promptly addressed (Neves et al.) (1). The risk of complications increases after 3 days of continuous use, making timely rotation of infusion sites a critical preventive measure (Neves et al.). Furthermore, environmental factors like temperature shifts and altitude changes can exacerbate these risks, causing malfunctions in insulin delivery (1). According to the study by Neves et al., addressing these challenges through comprehensive patient education on proper insertion techniques, regular site care, and monitoring is vital to improving outcomes and reducing adverse events in diabetes management serious adverse effects can occur even in the absence of an activated occlusion alarm, potentially disrupting insulin flow without warning.

Pomegranate peel has emerged as a promising source of bioactive compounds with significant anti-diabetic potential. Aslam et al. highlighted the peel's rich content of polyphenols, particularly tannins and flavonoids, which exhibit strong antioxidant and anti-inflammatory properties. In this study, authors used GC-MS based analysis of extracted compounds from *P. granatum* followed by application of computational algorithms to identify compounds with antioxidant, anti-inflammatory, and anti-diabetic properties. Using Auto Dock Vina software, they identified bioactive molecules extracted from *P. granatum* peel with high binding affinity for thioredoxin interacting protein (TXNIP), and key component of the thioredoxin system implicated in the regulation of glucose uptake in muscle (2). These compounds are associated with beneficial effects on glucose metabolism, insulin resistance, and β-cell function in T2D patients. Specifically, interactions between pomegranate peel metabolites and the TXNIP protein have been explored, revealing a potential mechanism for regulating oxidative stress and inflammation, key factors in diabetes management (Aslam et al.) (3). Further studies are needed to elucidate the precise molecular pathways, but the findings suggest that pomegranate peel could be developed into a functional food ingredient or therapeutic supplement for diabetes care (3).

The study titled: *Mendelian Randomization Analysis Demonstrates the Causal Effects of IGF Family Members in Diabetes* explores the complex roles of the insulin-like growth factor (IGF) family in diabetes, particularly in T1D and T2D. The findings highlight that elevated levels of IGF-1 increase the risk of T2D, while IGFBP-6 and adiponectin were protective. Conversely, IGF-1 and IGFBP-5 are associated with a reduced risk of T1D, while IGFBP-7 shows a positive correlation with T1D risk. These insights emphasize the intricate interplay between IGF family members and the pathogenesis of diabetes, offering new perspectives on potential therapeutic targets (Li et al.). In this meta-analysis, they found that GWAS datasets from diabetes along with circulating IGF subtypes uncovered pivotal association between IGF members with T1D and T2D via Mendelian randomization. This study also mentioned that major role of IGFs in developmental pathological in diabetes onset and urgent for experimental and observational study.

Recent advances in the management of GCK-MODY pregnancies highlight the critical role of precision medicine in enhancing fetal outcomes. Schwitzgebel et al. identified that a mutation in the glucokinase (GCK) gene is associated with maturity onset diabetes in the young (GCK-MODY) due to glucose impairment in pancreatic β-cells. GCK-MODY impacts glucose sensing in pancreatic β-cells, such that a fetus with the GCK mutation benefits from moderate maternal hyperglycemia, which supports fetal insulin secretion, while a wild-type fetus risks developing macrosomia under the same conditions. To address these variations, a non-invasive prenatal diagnostic (NIPD) test was developed to detect GCK mutations in the fetus using maternal plasma. This allows for tailored glycemic control, improving fetal outcomes without the risks of invasive procedures.

The article “*Identification of potential shared gene signatures between gastric cancer and T2D: a data-driven analysis*” reveals critical insights into the genetic links between gastric cancer (GC) and T2D (Xia et al.). By utilizing bioinformatics tools, researchers identified 104 overlapping genes that may play a role in both diseases. Key pathways, such as the “ECM-receptor interaction” and the “AGE-RAGE signaling in diabetic complications,” were highlighted as potential areas of crosstalk. Additionally, genes like BGN, VCAN, FN1, and COL1A1 were identified as potential therapeutic targets for individuals suffering from both GC and T2D, marking them as crucial for future research and precision medicine approaches.

Additionally, the integration of lifestyle interventions into precision medicine has garnered significant attention. Research demonstrates that personalized dietary and exercise regimens can lead to more effective glycemic control and weight management in individuals with diabetes. By tailoring these interventions to an individual's unique profile, healthcare providers can foster better adherence and outcomes. Another critical trend highlighted in this editorial is the use of technology and digital health tools. Continuous glucose monitoring (CGM), telemedicine, and mobile health applications are revolutionizing diabetes management. These technologies enable real-time monitoring and feedback, empowering patients to take an active role in their care. Moreover, data collected from these tools can be analyzed to refine precision medicine strategies further, allowing for timely adjustments based on individual patient responses.

Despite the promising advancements, several challenges remain in the implementation of precision medicine in diabetes. Access to genetic testing, the need for comprehensive training among healthcare professionals, and the ethical considerations surrounding data privacy and patient consent are critical issues that must be addressed. Additionally, there is a need for more extensive longitudinal studies to validate the long-term efficacy and safety of precision medicine approaches. In conclusion, the changing trends of precision medicine in diabetes represent a paradigm shift in how we approach the management and treatment of this complex disease. The manuscripts presented in this special issue provide valuable insights into the future of diabetes care, emphasizing the need for personalized strategies that enhance therapeutic outcomes and improve quality of life for patients. As we move forward, continued research and collaboration will be essential in realizing the full potential of precision medicine in combating diabetes.
